# Supplementary material for: Benchmarking of human read removal strategies for viral and microbial metagenomics
Source: Cell Rep Methods. 2025 Nov 5;5(11):101218. doi: 10.1016/j.crmeth.2025.101218 (PMC12664888; doi:10.1016/j.crmeth.2025.101218)
Supplement: Document S1. Figures S1–S4 and Tables S1–S3 [file mmc1.pdf]

Cell Reports Methods, Volume *5*

Supplemental information

Benchmarking of human read removal strategies

for viral and microbial metagenomics

Matthew Forbes, Duncan Y.K. Ng, Róisín M. Boggan, Andrea Frick-Kretschmer, Jillian Durham, Oliver Lorenz, Bruhad Dave, Florent Lassalle, Carol Scott, Josef Wagner, Adrianne Lignes, Fernanda Noaves, David K. Jackson, Kevin Howe, and Ewan M. Harrison

Method	False Negatives			False Positives			True Negative			True Positives		
	Max	Min	Median	Max	Min	Median	Max	Min	Median	Max	Min	Median
Bowtie2 VSL T2T-CHM13	28630	1	1978	375	0	5	7709249	1541	547612	7677739	1450	544607
Bowtie2 VSL GRCh38	30261	1	2083	373	0	3	7709279	1541	547612	7676133	1450	544494
MetaWrap-QC T2T-CHM13	37628	2	2565	156	0	3	7709303	1541	547612	7668855	1451	543973
Bowtie2-Kraken 2 T2T-CHM13	38630	2	2675	429	0	1	7708926	1541	547610	7667896	1450	543932
Bowtie2-Kraken 2 GRCh38	42512	2	2941	429	0	1	7708926	1541	547610	7664028	1449	543683
Bowtie2 Standard T2T-CHM13	57315	5	3960	49	0	0	7709338	1541	547612	7649382	1444	542679
SRA Human Scrubber	66637	6	4570	2	0	0	7709339	1541	547612	7640140	1449	542030
MetaWrap-QC GRCh38	72829	8	4884	159	0	2	7709307	1541	547612	7634132	1443	541723
Bowtie2 Standard GRCh38	111896	10	7524	49	0	0	7709338	1541	547612	7596221	1443	539187

Table S1: Maximum, minimum and median number of reads in each binary classification category across the synthetic mixture titration dataset. Rows are ordered by the Max False Negatives column. Related to Figure 2. Comparison of Human Read Removal Methods across the Synthetic Titration Dataset.

Alpha diversity metric	Raw (Bowtie2 Standard GRCh38)	SRA Human Scrubber	Bowtie2 VSL GRCh38	Bowtie2 VSL T2T-CHM13
**Richness**	31	19	11	11
**Shannon**	2.03	1.89	1.84	1.84
**Inverse Simpson**	5.06	4.77	4.84	4.81
**Pielou's evenness**	0.62	0.68	0.78	0.78

Table S2: Median calculation for all the alpha diversity. Related to Figure 3. Comparison of metagenomic outputs arising from metagenomics analysis post implementation of HRR methods.

Phylum	Bowtie2 VSL T2T-CHM13 vs Bowtie2 VSL GRCh38	Bowtie2 VSL T2T-CHM13 vs SRA Human Scrubber	Bowtie2 VSL T2T-CHM13 vs SRA Human Scrubber
**Actinomycetota**	-0.009	-0.192	-0.182
**Bacillota**	-0.002	-0.202	-0.199
**Bacillota_A**	-0.013	-0.167	-0.154
**Bacteroidota**	0.007	-1.021	-1.027
**Pseudomonadota**	0.006	-0.858	-0.864
**Spirochaetota**	0.014	-1.366	-1.38

Table S3: The delta mean of the beta coefficient obtained from a differential analysis. Related to Figure 3. Comparison of metagenomic outputs arising from metagenomics analysis post implementation of HRR methods.

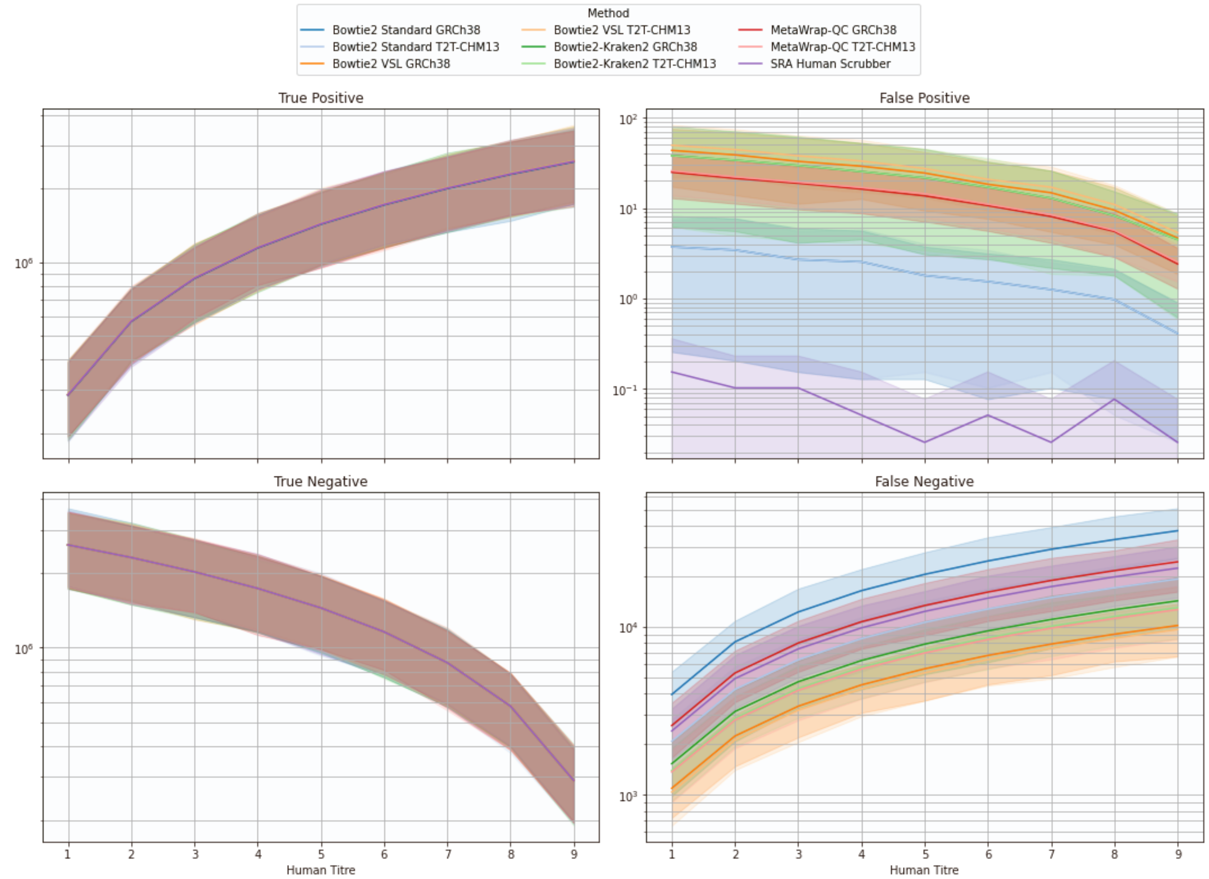

Fig. S1: Comparison of binary classifiers with regard to the Human Read Removal methods over the synthetic titration dataset. Lines show the mean value across all samples/iterations at a given human titre, shading shows the 95% confidence interval around the mean. Note

the different scales on the y-axis between plots when interpreting. Related to Figure 2. Comparison of Human Read Removal Methods across the Synthetic Titration Dataset.

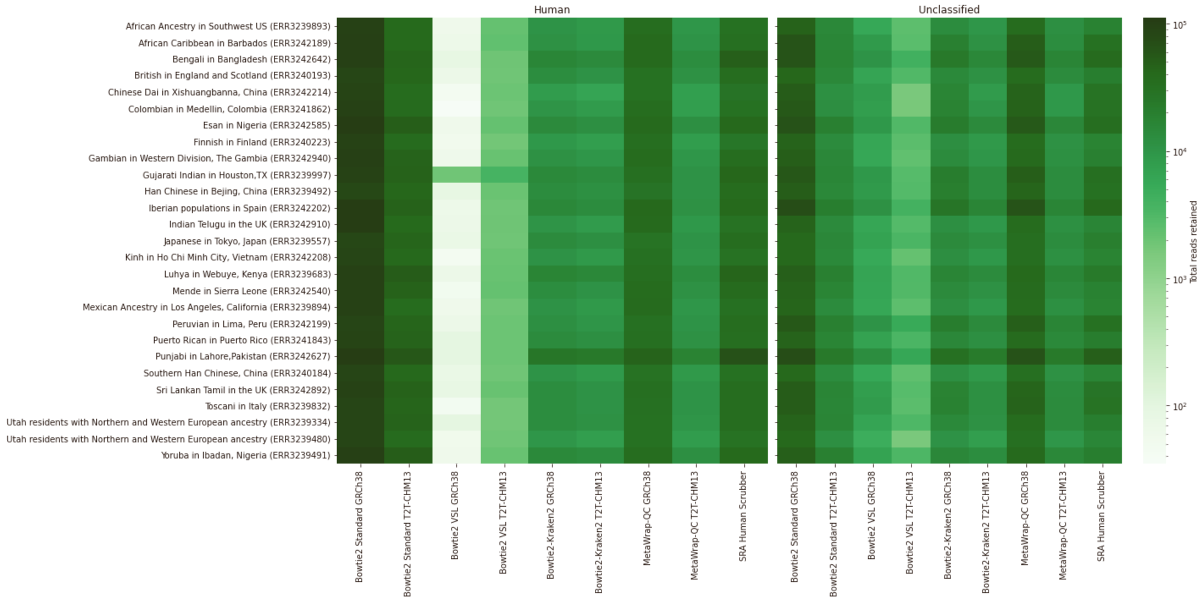

Fig. S2: Heatmaps showing the number of false negative reads (reads derived from human sources retained by HRR methods) which were classified by Kraken 2 as human (left plot) or were unclassified (right plot) per genetic ancestry. Related to Figure 2. Comparison of Human Read Removal Methods across the Synthetic Titration Dataset.

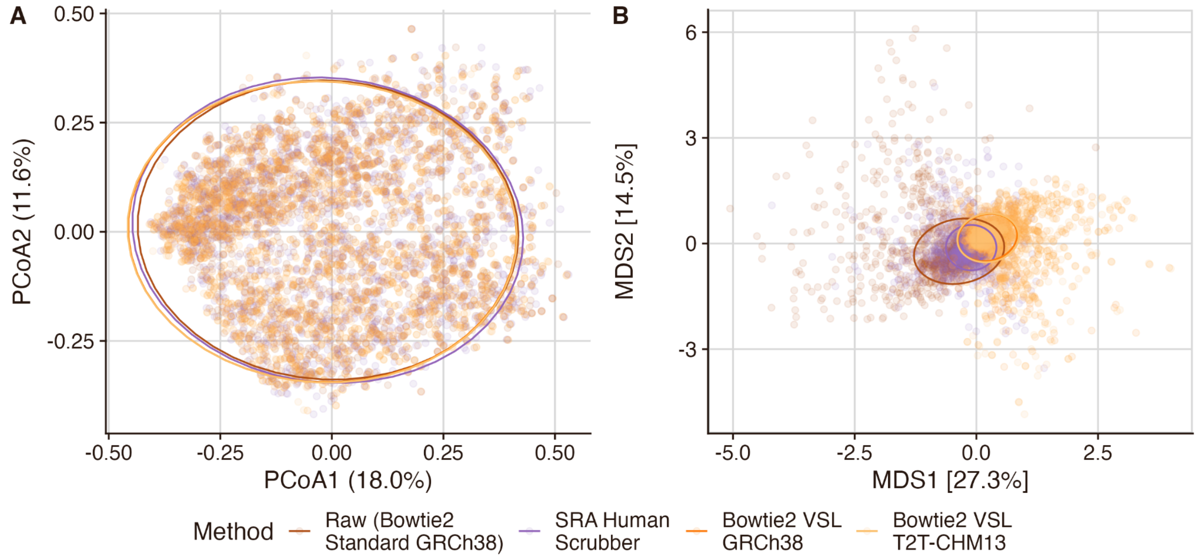

Fig. S3: Scatter plots representing betadiversity of microbial populations. (A) a PCoA of the metagenomic data coloured by the different HRR methods. (B) an MDS visualising the differences in the data after removing the variation contributed by the sample. Related to Figure 3. Comparison of Human Read Removal Methods across the Synthetic Titration Dataset.

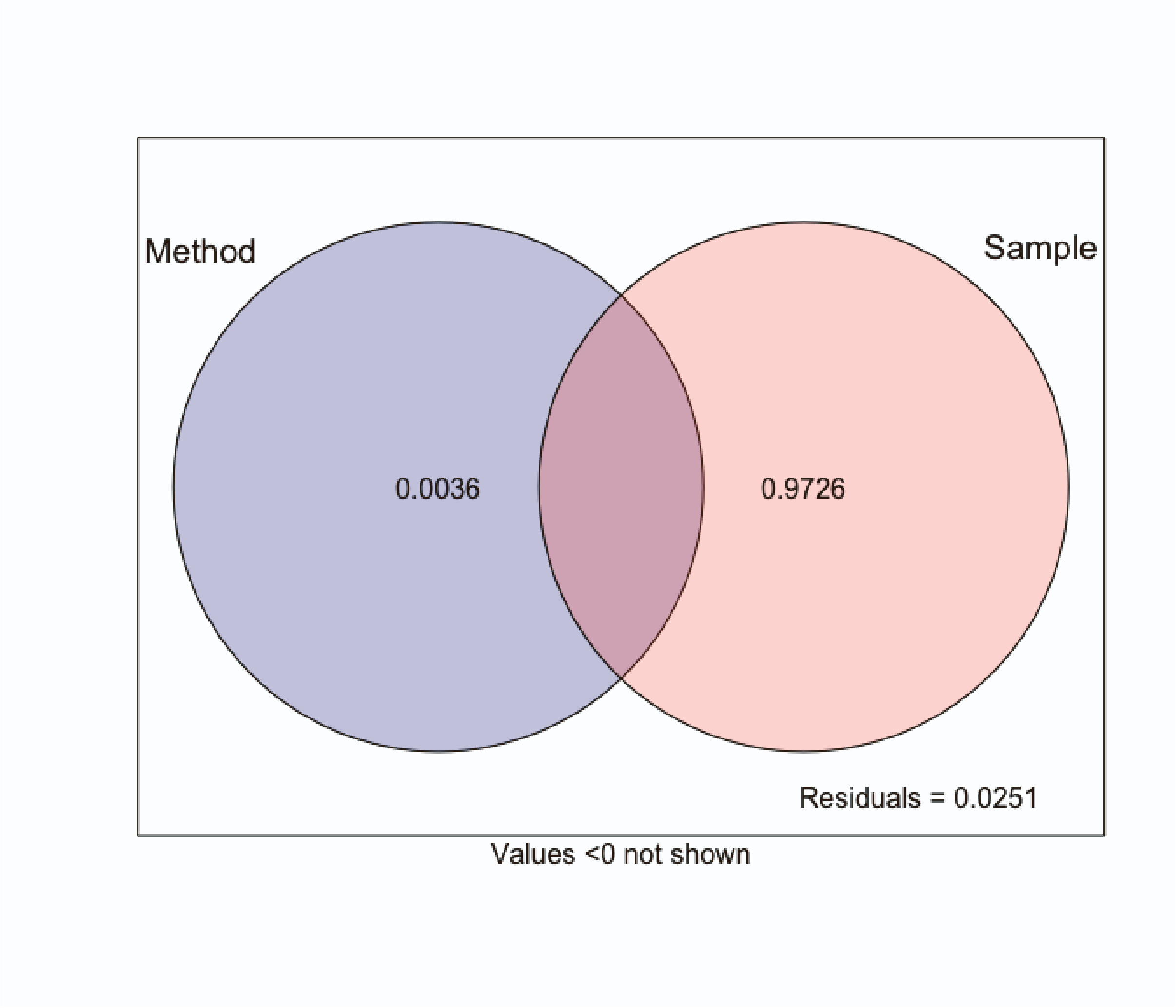

Fig. S4. A Venn diagram visualising the variation partition within the metagenomic dataset. Related to Figure 3. Comparison of Human Read Removal Methods across the Synthetic Titration Dataset.
